# Environmental Impact on DNA Methylation in the Germline: State of the Art and Gaps of Knowledge

**DOI:** 10.1155/2015/123484

**Published:** 2015-08-03

**Authors:** Francesca Pacchierotti, Marcello Spanò

**Affiliations:** Laboratory of Toxicology, Technical Unit of Radiation Biology and Human Health, CR Casaccia, ENEA, Via Anguillarese 301, 00123 Rome, Italy

## Abstract

The epigenome consists of chemical changes in DNA and chromatin that without modifying the DNA sequence modulate gene expression and cellular phenotype. The epigenome is highly plastic and reacts to changing external conditions with modifications that can be inherited to daughter cells and across generations. Whereas this innate plasticity allows for adaptation to a changing environment, it also implies the potential of epigenetic derailment leading to so-called epimutations. DNA methylation is the most studied epigenetic mark. DNA methylation changes have been associated with cancer, infertility, cardiovascular, respiratory, metabolic, immunologic, and neurodegenerative pathologies. Experiments in rodents demonstrate that exposure to a variety of chemical stressors, occurring during the prenatal or the adult life, may induce DNA methylation changes in germ cells, which may be transmitted across generations with phenotypic consequences. An increasing number of human biomonitoring studies show environmentally related DNA methylation changes mainly in blood leukocytes, whereas very few data have been so far collected on possible epigenetic changes induced in the germline, even by the analysis of easily accessible sperm. In this paper, we review the state of the art on factors impinging on DNA methylation in the germline, highlight gaps of knowledge, and propose priorities for future studies.

## 1. Introduction

Epigenetics formally refers to heritable changes in gene expression and in phenotype occurring without changes in the underlying DNA sequence. Alterations in epigenetic marks have been involved in the etiology of complex syndromes and diseases, including cancer, infertility, cardiovascular, respiratory, metabolic, immunologic, and neurodegenerative pathologies [1–3]. The main epigenetic mechanisms responsible for these alterations are represented by DNA methylation, posttranslational histone modifications, and regulation by noncoding microRNAs [4]. In particular, DNA methylation, the most studied epigenetic mark so far, involves the enzymatically mediated covalent addition of a methyl group to the C5 position of cytosine, forming 5-methyl cytosine (5-mC). Cytosine methylation primarily happens in CpG dinucleotides in CpG-rich sequences, dubbed as CpG islands, often occurring near or in the gene promoter regions. DNA methylation, operated by a family of DNA methyltransferases [5], is implicated in many life-essential cellular and developmental processes, such as embryonic reprogramming, cellular differentiation, silencing of genes and transposons, parental imprinting, X chromosome inactivation, and genomic stability [6–8].

It is largely accepted that exposure to a variety of environmental toxicants has a negative impact on human health and contributes to the development of a large array of diseases. The epigenome is more plastic and flexible than the genome. Changes of epigenetic marks, such as DNA methylation, can affect the chromatin structure and modify binding of transcription factors and gene expression. The theoretical framework of a changing environment and a modifiable epigenome might offer unexplored and unsuspected ways to understand gene-environment interactions and potentially mitigate the impact of environmental toxicants on human health [9, 10].

A rapidly growing number of epidemiological studies have been carried out throughout the world, in which environmental exposure and lifestyle (an umbrella term including diet, behavior, stress, physical activity, working habits, voluntary alcohol and tobacco consumption, etc.), meshed with the genetic background, have been associated with epigenetic changes, mostly DNA methylation (reviewed in [11–18]). The trend in the field appears to shift from the introduction of a novel “proof-of-principle” approach in toxicology to a more systematic scientific specialty [19, 20].

Environmental epidemiology research is addressing epigenetic mechanisms as mediators of environmental exposure on disease risk or just as biosensors of exposure even if not mechanistically relevant. Because stable methylation marks at differentially methylated regions (DMRs) regulating imprinted genes are acquired before gastrulation, they may serve as archives of early exposure with the potential to improve our understanding of developmental origin of adult diseases.

DNA methylation has been by far the most extensively measured epigenetic mark because of its obviously fundamental biological interest, its mitotic stability, the availability of methods for its quantification, globally or in targeted regions, its stability during the DNA extraction and purification procedures, and its durability in archival biological materials. By and large, the strategy consists of comparatively assessing the methylation differences at CpG islands in gene promoters or DMRs, between control and exposed groups.

The information on DNA methylation status and changes in association with environmental exposure and lifestyle has been mostly collected from peripheral blood leukocytes (PBL), which can be sampled by a minimally invasive approach. However, tissue specificity, together with purity of cells for DNA methylation determination, represents a relevant issue in epigenetic studies as each tissue and, within a tissue, probably each cell type have its own epigenetic profile.

During our lifetime, the genome undergoes two main epigenomic reprogramming periods, each of which involves waves of DNA demethylation and de novo methylation. These precise and coordinated genome-wide reprogramming steps are associated with pivotal developmental stages like the establishment of cell totipotency and the differentiation of the germ cell lineages [21, 22]. The first wave occurs, with notable differences between sexes, in all cells of the preimplantation embryo. The second wave occurs in primordial germ cells (PGCs) only; this time the demethylation events are more radical and involve imprinted genes whose allelic-specific methylation is first erased and then reset according to the sex of the germline [23]. Conceivably, these phases of mammalian development are especially sensitive to environmental stressors, which can impact epigenetic plasticity with potential enduring effects on metabolic pathways and disease susceptibility. Indeed, such scenario would be in agreement with the theory of the fetal basis of adult onset disease [24–26].

The early fetal period of life is particularly critical for gonadal development, and many common reproductive disorders of the adult male, such as infertility and testis cancer, have been proposed to have a fetal origin [27]. In addition, prenatal exposure to environmental contaminants, especially those belonging to the variegated and heterogeneous class of compounds collectively defined as endocrine disruptors (EDs), has been linked to the increased incidence of male reproductive pathologies [28–32]. The interference with developmental epigenetic processes has been evoked as one of the potential mechanisms of EDs action affecting the integrity of the male reproductive system [33]. Recent evidence that unbalanced one carbon metabolism may impact male reproductive health [34] and that a variety of epigenetic markers, including global or gene-specific DNA methylation, can be altered in infertility patients [35, 36] is in agreement with this hypothesis.

In spite of the extensive DNA demethylation occurring in preimplantation embryonic cells, there are sequences, corresponding primarily, but not exclusively, to parentally imprinted genes that escape global demethylation [37, 38]. This means that changes of DNA methylation induced by environmental stressors in germ cells could not only have consequences for the reproductive health of the exposed individual but also might be potentially heritable from one generation to another and might cause transgenerational adverse effects by a nongenetic mechanism of inheritance.

Notwithstanding the knowledge about epigenetic regulation of gonadal development and the evidence about epigenetic changes induced in rodent germline by several chemicals or dietary conditions, the number of studies aimed at testing possible effects of lifestyle or chemical exposure on human sperm DNA methylation is extremely limited in comparison to the number of studies carried out on blood cells. Sperm can be obtained by a similarly noninvasive procedure; they represent the target cell for male reproductive effects and not merely a surrogate of it; their ultimate DNA methylation pattern is acquired by a multistep process started in PGCs and completed during the spermatogonia and spermatocyte differentiation phases [39, 40], which might therefore be repeatedly exposed to environmental insults. For all these reasons it would be very valuable to extend the analysis of sperm epigenetics beyond infertility clinical investigation to environmental biomonitoring studies.

Focus of this literature review will be on data linking various exogenous factors, from specific chemical exposure to psychological stress, to DNA methylation changes in the germline and their consequences in the offspring. We have taken into consideration both experimental rodent and human studies. In addition, due to the very limited amount of data from human biomonitoring investigations, we have decided to include a survey of papers reporting human population studies which have shown an environmental impact on DNA methylation of somatic cells, to highlight those sources of exposure that would be worth further germline-oriented investigations, and the present gaps of knowledge.

## 2. Human Studies on Environmentally Linked DNA Methylation Changes in Somatic and Germ Cells

Several recent and excellent reviews [12, 13, 15, 17, 41, 42] have been published on this subject. Here, we try to offer an original aggregation pattern of the data, which are mostly very recent but often incomplete and methodologically heterogeneous, to highlight research trends and gaps of knowledge, also in relation to experimental rodent studies. The epidemiological studies specifically reporting the effects of environmental chemicals on DNA methylation are summarized in Table 1.

### 2.1. Metals: Arsenic, Chromium, Cadmium, Lead, Mercury, and Selenium

Environmental toxic metals have been associated with important human pathologies like cancer, cardiovascular and autoimmune diseases, and neurological disorders and, recently, their impact on the epigenome has started to be explored [43].

Inorganic arsenic is a carcinogenic metal. Several millions of people around the world are exposed to arsenic concentrations in their drinking water that exceed the World Health Organization's recommended limit of 10 ppb. The mechanism(s) of arsenic toxicity and carcinogenicity are not fully clarified; recently, epigenetic alterations have been proposed to play a role and have been explored in cohort and case-control studies, especially in Asian populations living in highly contaminated areas (reviewed in [44]). Exposure, generally assessed by the metal concentration in drinking water and/or in biological fluids or tissues, has been associated with dose-dependent global DNA hypermethylation [45–47] and with hypermethylation of specific oncosuppressor genes [48–52]. Genome-wide comparisons of DNA methylation patterns from people who developed skin lesion and a control group in Bangladesh have evidenced 6 CpG sites with greatest changes of DNA methylation among cases, one of which belongs to the* RHBDF1* gene, previously reported to be hypermethylated in arsenic-exposed cases [53]. Similarly, by using high throughput approaches, specific DNA methylation changes in particular genes were detected between arsenic-induced and non-arsenic-induced urothelial carcinomas in Taiwan [54].

The epigenetic effects of in utero arsenic exposure were investigated in umbilical cord blood to find out a mechanistic basis for possible arsenic-induced alterations of fetal developmental programming. Hypermethylation of the transposonic repeat* LINE-1*,* p16* promoter, and other specific sequences was associated with arsenic concentration in maternal drinking water [55, 56]. Other studies also showed some effects of arsenic maternal exposure on cord blood DNA methylation [57, 58], although the involved sequences were not always consistent.

The newborn blood DNA methylation pattern seems to be affected also by in utero exposure to low concentrations, as shown by the results of a prospective American birth cohort study using high throughput arrays [59]. In another large Mexican cohort, a total of 2,705 genes in cord blood leukocytes showed differences in DNA methylation that were associated with maternal exposure to arsenic in drinking water. The gene set was highly enriched in binding sites of the early growth response and CTCF transcription factors. Furthermore, DNA methylation levels of seven of these genes were associated with differences in birth outcomes including gestational age, placental weight, and head circumference [60]. These results strongly point to the need for long-term follow-ups to determine whether the observed DNA methylation changes may be associated with specific health outcomes.

Chromium VI [61], mercury [62, 63], lead [64, 65], cadmium [62, 66], and selenium [67] are other metals for which association studies between human exposure and DNA methylation changes, mainly in peripheral blood cells, have been conducted. Cadmium can cross the placental barrier and its potential as a developmental toxicant has been studied in an American survey by comparing maternal blood cadmium levels during pregnancy and genome-wide DNA methylation in leukocyte DNA collected from cord blood cells [68]. A variety of genes showed methylation changes associated with maternal cadmium concentrations. The set was enriched in genes involved in transcriptional regulation control and apoptosis. Conserved DNA motifs with sequence similarity to specific transcription factor binding sites were identified within the CpG islands of the gene set. Altogether the results pointed to a possible functional impact of cadmium on fetal DNA methylation.

Overall, the number of studies on the epigenetic impact of environmental metal exposure is limited. The study designs, the number of people enrolled, the genomic sequences investigated, and the methods used to assess methylation changes (locus-specific, global locus-independent, epigenome-wide) are quite heterogeneous. Widely different exposure levels have been evaluated in occupational studies and in studies on the general population. Therefore, any attempt to draw general conclusions is still premature. However, the expected decrease of costs of epigenome-wide analytical methods will likely allow acquiring a wealth of data in the near future. In addition to such unsupervised studies, more focused investigations on global hypomethylation and downregulation of the methylation machinery, hypomethylation in regions controlling transposons or oncogene expression, or hypermethylation at oncosuppressor genes could offer the best contribution to unravel epigenetic mechanisms underlying environmental cancer and to develop novel predictive biomarkers.

### 2.2. Air Pollution (Particulate Matter, Polycyclic Aromatic Hydrocarbons, Benzene, and Volatile Organic Compounds)

Exposure to air pollution is a side-product of urbanization and industrialization representing a dramatic health problem, associated with childhood asthma, wheeze, and increased cardiovascular morbidity and mortality. It is generally assessed by measuring the levels of particulate matter with aerodynamic diameter ≤2.5 (PM2.5) or ≤10 *μ*m (PM10) together with the levels of other air pollutants like black carbon, ozone, polycyclic aromatic hydrocarbons (PAHs), sulfur, and nitrogen dioxide. There have been several studies carried out across the globe, which have considered possible impacts of air pollution on DNA methylation with sometime contrasting results [69].

In a recent European study on a cohort of young nonsmoking subjects, the exposure to ambient concentrations of NO_2_, PM10, PM2.5, and O_3_ and traffic parameters were associated with a decreased global DNA methylation level in blood cells [70].

Numerous studies focused on the vulnerable subpopulation of elderly people [71–74]. Hypomethylation of repeated sequences (*LINE-1* and, in some cases, also* Alu*) was reported to be associated with increased pollutant concentrations. In addition, methylation changes of specific genes involved in inflammatory and immune response pathways were observed, which were regarded as modifiers of the association between air pollutants and reduced lung function [74].

Other studies investigated effects of air pollution on DNA methylation in children, showing changes in genes involved in asthma morbidity [75] or nitric oxide metabolism in airways [76, 77].

Lower global DNA methylation [78] and hypermethylation of specific genes [79, 80] in umbilical cord white blood cells were shown to be associated with maternal exposure to airborne PAHs, pointing to a possible prenatal environmental epigenetic origin of childhood diseases.

Workers of different job sectors, exposed to particulate matter, PAHs, and benzene, have been also monitored for possible DNA methylation changes in peripheral blood cells [81–91]. In general, DNA repetitive elements, such as* LINE-1*,* Alu*, and* HERV*, have been analyzed, in addition to specific genes including* p53*,* p15*,* p16*,* APC*,* RASSF1A*,* HIC1*,* iNOS*,* hTERT*, and* IL-6*. Each specific gene and subfamily of repetitive sequences seem to respond independently to the exposure and no set of sequences has yet emerged as an ideal reporting system of epigenetic effects. In addition, exposure conditions and methods of assessment were quite heterogeneous making any overall conclusion impossible. These studies support the notion that epigenetic biomonitoring is still a new area of environmental health studies that necessitates of international coordination, methodological harmonization, and mechanistically sound interpretation of results.

### 2.3. Persistent Organic Pollutants (POPs) and Endocrine Disruptors (EDs)

This heterogeneous class of chemicals is strongly suspected to interfere with the human hormonal homeostasis and to hamper reproductive integrity, especially when exposure occurs during the pre- and perinatal life stages. In a cohort of Greenland Inuits, in DNA extracted from blood samples,* Alu* sequences showed significant hypomethylation as a function of increasing blood concentration of p,p′-DDT [1,1,1-trichloro-2,2-bis(p-chlorophenyl)ethane], its main metabolites p,p′-DDE [1,1-dichloro-2,2-bis(p-chlorophenyl)ethylene], *β*-HCH (hexachlorocyclohexane), oxy- and *α*-chlordane, mirex, sum of PCBs (polychlorinated biphenyls), and sum of POPs; the methylation level of* LINE-1* sequences showed a similar inverse trend with the exposure, albeit not statistically significant [92]. In agreement with these data, the blood concentrations of various organochlorine pesticides in a cohort of healthy Koreans were inversely associated with the methylation level of* Alu* but not of* LINE-1* sequences [93].

Another widely debated chemical is the ubiquitous bisphenol A (BPA), a monomer used in epoxy resins and polycarbonate plastics. Exposure to BPA and possible methylation alterations were studied with genome-wide approaches. Significant hypomethylation of the* TSP50* gene promoter in blood cells was associated with BPA exposure in a cohort of women undergoing in vitro fertilization (IVF) [62]. In a survey of prepubescent Egyptian girls [94] higher urinary BPA concentrations were associated with lower genomic methylation and, interestingly, many affected genes were among those whose expression changes had been previously associated with BPA exposure.

Another class of emerging POPs is represented by perfluoroalkyl substances (PFASs), which include a variety of compounds widely used in many industrial processes and products. Cross-sectional associations between serum PFASs and* LINE-1* DNA methylation were evaluated in an American population highly exposed via contaminated drinking water [95]. A significant association was found for some but not all specific PFASs. To explore the possible effects on male reproduction, global methylation and* LINE-1, Alu*, and* Satα* methylation levels were directly assessed in sperm DNA from fertile men from Greenland, Poland, and Ukraine characterized by a wide contrast to PFASs plasma levels. No strong consistent associations between PFASs exposure and any of the sperm methylation biomarkers could be detected [96].

Three studies explored the influence of maternal POPs serum concentrations on DNA methylation of umbilical cord blood cells. Global DNA hypomethylation appeared to be associated with the serum level of specific PFASs [97]. Interestingly, two studies examining the effects on various families of repeated DNA sequences [98, 99] showed that the association between serum xenoestrogen contamination and* Alu* hypomethylation in cord blood was influenced by the baby gender, in agreement with the hypothesis of a differential, gender-dependent, susceptibility to prenatal EDs exposure.

### 2.4. Antibiotics

Low birth weight (LBW) has been associated with common adult-onset chronic diseases. Its etiology is multifactorial and exposure to antibiotics has been reported to increase LBW risk. Among possible mechanisms underlying this association, epigenetic changes have been proposed.

In the US Newborn Epigenetics Study (NEST), the methylation status of the DMRs of a variety of growth regulatory imprinted genes (*IGF2, H19, MEST, PEG3, PLAGL1, SGCE/PEG10, NNAT,* and* MEG3*) was analyzed, in umbilical cord blood cells, in relation to the infant birth weight and maternal (self-reported) antibiotic use. Methylation at* IGF2*,* H19*,* PLAGL1*,* MEG3*, and* PEG3* was associated with maternal antibiotic use, although only methylation at the PLAGL1 DMR was also associated with birth weight [100].

### 2.5. Tobacco Smoke

The potential epigenetic links between current and prenatal smoking and smoking-related diseases are extensively discussed in recent review papers [101, 102]. Smokers and nonsmokers have been compared by high throughput methods in several cohorts of adult and young people, with some consistent alterations detected involving DNA methylation differences at specific positions in the* F2RL3* [103–107], in the* AHRR* [108–112], and in* GPR15* genes [112], which emerged as strong candidates to predict smoking-related negative health outcomes. Epigenetic changes in the offspring of mothers smoking during pregnancy have been characterized by genome wide approaches, to contribute unraveling the mechanistic pathways of some, well-known, prenatal smoking-related adverse effects. Accumulating data indicate that prenatal exposure to tobacco smoke is associated with reproducible epigenetic changes at a global and gene-specific level that persist well in childhood and adolescence [97, 113–122]. Changes have been found, among others, in genes involved in transcription, in oxidative stress and detoxification pathways, and in repetitive elements, even though the biological significance of a variety of altered loci remains to be understood.

### 2.6. Parental Influence

#### 2.6.1. Paternal Effects

In humans, there is sparse evidence linking lifestyle paternal factors with the offspring epigenome [123]. Paternal obesity has been associated with hypomethylation at the* IGF2* [124] and* MEST*,* PEG3* and* NNAT* [125] DMRs in the offspring cord blood cells, independently of maternal obesity and other potential confounders.

It is noteworthy that global sperm DNA methylation has been shown to increase, on average, by 1.76% per year [126], and an in-depth analysis of the methylome in two sperm samples collected 9–19 years apart from 17 fertile American men has shown several age-related changes [127]. One hundred and thirty-nine regions were significantly and consistently hypomethylated and 8 regions were significantly hypermethylated with age; 117 genes were associated with these regions of methylation alterations (promoter or gene body), with a portion of them surprisingly located at genes previously associated with schizophrenia and bipolar disorder. In the same samples,* LINE-1* showed global hypermethylation with age, while another study, aimed at relating numerous variables with sperm DNA methylation [128], did not show age-dependent changes in* LINE-1*,* Alu*, and* Satα* methylation level, probably because of the narrow age contrast of studied populations. In the latter study, personal characteristics and habits, body mass index (BMI), semen quality parameters, sperm chromatin integrity, biomarkers of accessory gland function, and the plasma concentration of reproductive hormones were related to sperm DNA methylation in a cohort of 224 men of proven fertility, living in three European regions, Greenland, Warsaw, and Kharkiv. The geographical location emerged as the main determinant of the methylation level in repetitive sequences and no other consistent associations between methylation markers and the assessed variables were identified across countries [128].

Until now, only three human biomonitoring studies addressed the impact of environmental factors on sperm DNA methylation. One is the already cited investigation on the possible effects of PFASs exposure on* LINE-1*,* Alu*, and* Satα* methylation level [96], which did not show consistent PFASs-associated alterations. The other two studies focused on occupational radiation exposure and alcohol consumption. An increase of hypermethylated spermatozoa was shown in radiation-exposed workers [129]. Some years ago, alcohol had been shown to reduce the methyltransferase mRNA levels in sperm of chronically treated rats with potential consequences on paternal imprinting [130]. Notably, a trend of increased demethylation with alcohol consumption was shown, in sperm of male volunteers, at the* H19* and* IG* DMRs, with a significant difference observed at the* IG*-DMR between the nondrinkers and heavy alcohol consumers [131].

#### 2.6.2. Maternal Effects

Newborn methylomes contain molecular memory of the individual in utero experience [132]. In the above chapters we have discussed various examples linking maternal environmental exposure to newborn DNA methylation, as assessed through cord blood cell analysis. However, the maternal impact appears to extend beyond that of specific chemical contaminants, as also metabolism, nutrition, and stress seem to influence the offspring methylome.

In an American black mother-child cohort study, genome-wide analysis of cord blood cells showed that about 20 CpG sites in cancer and cardiovascular disease relevant genes were highly significantly associated with maternal BMI [133].

The epigenetic consequences of prenatal famine and caloric restriction have been evaluated in a cohort of people conceived in the winter 1944-45 during a severe famine at the end of World War II (Dutch Hunger Winter Families Study). These people appear to bear the consequences of prenatal stress later in life, including an adverse metabolic profile (suboptimal glucose handling, higher BMI, and elevated total and low-density lipoprotein cholesterol) and increased risk of schizophrenia. While the overall global methylation levels in their blood cells appear to be unaffected [134], significant DNA methylation changes have been shown, at several specific loci corresponding to imprinted genes or to genes implicated in growth and metabolic diseases, including* IGF2, IL-10, LEP, ABCA1, GNASAS*, and* MEG3* [135–139]. A genome-scale analysis has demonstrated that differential DNA methylation preferentially occurs at regulatory regions and maps to genes enriched for differential expression during early development [140]. Changes have been also shown to depend on the sex of the exposed individual and the gestational timing of the exposure [137].

Folate plays an essential role in one-carbon metabolism involving remethylation of homocysteine to methionine, which is a precursor of* S-*adenosylmethionine, the primary methyl group donor for most biological methylations, including DNA methylation. A few pilot studies considered possible effects of maternal intake of methyl-donor compounds on their infant DNA methylation. Compared to infants born to women reporting no folic acid intake before or during pregnancy, methylation levels at the* H19* DMR in umbilical cord blood leukocytes decreased with increasing folic acid intake, the decrease most pronounced in the male offspring [141]. In another study [142], increased methylation at the maternally* IGF2* imprinted gene and decreased methylation at the maternally imprinted gene* PEG3* and at the repetitive transposonic sequence* LINE-1* were associated with folic acid supplementation after the 12th week of gestation but not during the first trimester or before conception. Finally, a third study [143] did not detect any major association between intake of methyl donor nutrients (vitamin B12, betaine, choline, folate, Cd, Zn, and Fe) during pregnancy and* LINE-1* DNA methylation.

The results of a human epidemiological study, conducted in rural populations in Gambia experiencing pronounced seasonal fluctuations in nutritional status and diet, support a role for periconceptional maternal plasma concentration of key micronutrients involved in one-carbon metabolism on infant DNA methylation [144, 145]. The study focused on the analysis of human candidate metastable epiallelic loci [25, 146]. Metastable epialleles are genomic regions where epigenetic patterning occurs before gastrulation in a stochastic fashion leading to systematic interindividual variation within one species. Their existence is well documented in the mouse since the pioneering studies on the* agouti viable yellow* (*A* ^*vy*^) locus, and, in this model, maternal diet has been shown to modulate the establishment of the epigenetic marks (reviewed in [38]). The human survey has shown that DNA methylation at metastable epialleles in lymphocytes and hair follicle cells of infants conceived during the rainy (“hungry”) season is significantly different from that of infants conceived in the dry (“harvest”) season, providing first evidences of a lasting and systemic effect of periconceptional environment on human epigenotype.

Maternal depression has been associated with a higher risk of LBW and hypermethylation at the* MEG3* DMR of infants [147]. Furthermore, LBW infants had lower methylation at the* IGF2* DMR, while high birth weight infants had higher methylation at the* PLAGL1* DMR compared with normal birth weight infants. Thus, imprinted gene plasticity may play a role in the observed association between depressive mood in pregnancy and LBW.

Preliminary human studies are providing first evidence supporting the conclusion that traumatic experiences can result in lasting, broad, and functionally organized DNA methylation signature in several tissues in offspring. A Canadian study (Project Ice Storm) was set up some months after the 1998 Quebec ice storm by recruiting women who had been pregnant during the disaster, scoring their degrees of objective hardship and subjective distress. Thirteen years later, genome-wide DNA methylation profiling in T cells obtained from 36 of the children was assessed. Prenatal maternal objective hardship (but not maternal subjective distress) was correlated with DNA methylation levels in 1675 CpGs affiliated with 957 genes predominantly related to immune function [148].

## 3. Rodent Experimental Studies on the Induction of Epigenetic Changes in the Germline

In the last few years, experiments in rodents started to test the hypothesis that exogenous exposure to some measurable factor, during a controlled time window, could induce epigenetic changes in the germline. The large majority of these experiments investigated changes of DNA methylation at a global, gene-specific, or genome-wide level and will be discussed in this section. A few considered also other types of epigenetic changes such as the sperm microRNA content [149–151], but, due to their still very small number, they will not be further addressed.

These studies were prompted by the observations of heritable traits unexplained by Mendelian inheritance. However, only a subset of studies showing epigenetic transgenerational effects also provided evidence of potentially heritable epigenetic changes in the exposed gametes. In this review, only those studies that analysed possible DNA methylation changes in the male or female germline of exposed animals have been considered.

Overall, 24 papers were reviewed, 19 reporting studies in mice and 5 in rats. Studies on the possible induction of epigenetic alterations in germ cells were grouped according to the exposure time window: either prenatally, during the critical period of germline differentiation (10 studies), or postnatally, in prepuberal or adult male (12 studies) or female (5 studies) animals. One of the 5 studies on exposure of the female germline was carried out in vitro (Table 2).

From the emerging overview, the research objectives still appear rather sparse: a group of studies evaluated the impact of metabolic changes, due to undernourishment [152], low-protein [153], folate-deficient [154], zinc-deficient [155], obesogenic, and/or diabetogenic diets [149, 156–158]. Other studies investigated the effects of specific compounds, many of which belong to the class of so-called endocrine disrupters, including vinclozolin [159–161], methoxychlor [162], dioxin [163], and bisphenol A [164–166]. The remaining studies deal with a heterogeneous group of potentially epigenetics disrupting agents: particulate air pollution [167], ethanol [168, 169], tamoxifen [170, 171], fenvalerate [172], and sodium fluoride [173].

Regarding the genomic targets, several studies focused on a few loci, either maternally (*Mest, Snrpn, Igf2r*, and* Peg3*) or paternally (*H19, Meg3,* and* Rasgrf1*) imprinted (methylated). Other studies evaluated changes of methylation in metabolism-related genes, such as the* LPLase*, the* Pparα*, or the* Lep* gene. One paper included the analysis of methylation of* Line-1* repeated sequences [173]. A few studies assessed possible changes of total DNA methylation, whereas the most recent papers report analyses at a genome-wide level. With a few exceptions [153, 160, 163, 170, 173], the studies showed some kind of exposure-related effect. Both increase and decrease of methylation levels were reported. As pointed out before, the studies are too scattered and too heterogeneous in the analytical methods to allow drawing general conclusions; however, some hints are emerging, like increased methylation of maternally imprinted and decreased methylation of paternally imprinted genes in the exposed male germline. Interestingly, the few studies on oocyte exposure show an opposite effect of treatment on the same maternally imprinted genes, which seem to respond with a decreased methylation level.

In some studies the impact of exposure on germ cells has been compared with the impact on somatic cells. Depending on the type and time of exposure, some studies showed that the methylation control mechanisms were more robust in the somatic than in the germ cells, as in the case of prenatal treatment with ethanol [168] or methoxychlor [162], but a reversed sensitivity was also observed, as in the case of prenatal exposure to dioxin [163]. It is conceivable that each tissue might respond accordingly to its specific developmental program; therefore, studies of exposure during the period of prenatal germline differentiation are especially important for assessing any environmental epigenetic impact on the germline.

The evaluation of an epigenetic impact of exogenous factors on the germline is still in its infancy. A critical issue that has not yet been thoroughly addressed is if and how much the DNA methylation changes have a functional impact on the gene expression level and have any causal role on the male germ cell toxicity that is sometimes induced, as after prenatal vinclozolin [161] or ethanol [168] exposure.

A group of studies aimed mainly to evaluate possible transgenerational consequences of epigenetic alterations in the germline. The simplest hypothesis was that methylation changes in gametes could resist zygotic reprogramming and have functional consequences in the offspring sired by exposed animals.

Indeed, in mice, ethanol exposure in utero was shown to induce* H19* demethylation in sperm of adults, as well as in the brain cells of their offspring, with a good concordance between the CpG demethylation patterns across cell types and generations [168]. In another study testing possible transgenerational effects of tamoxifen in rats [171, 174], the offspring sired by tamoxifen-treated animals showed an increased incidence of embryonic resorptions, and resorbed embryos (but not normal ones) carried methylation errors similar to those detected in the sperm of exposed fathers. In male mice a prediabetic condition closely resembling the metabolic abnormalities of human prediabetes can be induced by high-fat diet and chemical treatment; these mice transmit to their offspring glucose intolerance and insulin resistance [156]. Epigenomic profiling in offspring pancreatic islets identified changes in cytosine methylation at several insulin signaling genes, and these changes correlated with the expression of these genes. The analysis of cytosine methylation profiles in sperm of prediabetic fathers showed several alterations and a large proportion of differentially methylated genes overlapped with that of the offspring pancreatic islets. Bisulfite sequencing of some of these genes in blastocysts showed that they resisted global postfertilization demethylation and largely inherited cytosine methylation from sperm, suggesting that there might be intergenerational transmission of methylation profiles.

However, other studies demonstrated that epigenetic inheritance via the gametes can be more complex than the direct transmission of DNA methylation alterations, and a crosstalk might exist between different levels of epigenetic regulation across generations.

A genome-wide analysis of methylation changes in sperm of mice exposed to a folate-deficient diet showed altered methylation profiles in genes implicated in development, chronic diseases such as cancer, diabetes, autism, and schizophrenia [154]. In the same study, a twofold greater resorption rate and an increased frequency of developmental abnormalities were observed in pregnancies sired by exposed males. Moreover, significant changes in the expression of over 300 genes were detected in the placenta of exposed-animals sired offspring. However, differentially expressed genes in the placenta did not match differentially methylated genes in sperm, suggesting that mechanisms other than DNA methylation might be involved in the transgenerational transmission of epigenetic messages, like sperm histone modifications.

Similarly, in utero undernourishment induced hypomethylation of several genes in sperm, as well as changes of expression of metabolic genes in the brain and liver of late gestation fetuses sired by the exposed animals; interestingly, the genes whose expression was altered in the fetuses mapped close to differentially methylated regions in sperm, although differential methylation was not transgenerationally retained [152]. The authors concluded that “… it is unlikely that these expression changes are directly mediated by altered methylation; rather, the cumulative effects of dysregulated epigenetic patterns earlier in development may yield sustained alterations in chromatin architecture, transcriptional regulatory networks, cell type, or tissue structure.”

Postweaning growth delay and decreased methylation at the* H19* ICR* CTCF* binding sites were observed in the offspring of adult mice treated with ethanol, although no decrease of* H19* DNA methylation was detectable in the sperm DNA [169]. Methylation was significantly increased in* Peg3* and significantly decreased in* H19* 8-cell embryos sired by male mice treated with sodium fluoride, while no change of methylation was detected in sperm [173]. Increased methylation of a number of imprinted genes, associated with downregulation of transcription, was detected in the resorbing embryos sired by tamoxifen-treated male rats, in spite of the fact that their sperm did not show DNA methylation changes in any of the 9 analyzed imprinted genes [170].

The complexity of the interplay between environmentally sensitive epigenetic markers in sperm and epigenetic modulation of development in the following generation is further illustrated by a recently published report on the transgenerational consequence of paternal exposure to a conditioning olfactory experience [175]. The F1 progeny of conditioned mice reacted just like the fathers with enhanced response, in spite of never being conditioned themselves. The F1 neuroanatomy was also affected. Behavioral sensitivity and neuroanatomical alterations in the nervous system were present also in the IVF-derived F1 generation and persisted until at least the F2 generation. Hypomethylation in specific CpG islands of the* Olfr151* gene encoding for the specific odor receptor was detected in sperm of exposed mice. These findings led the authors “to hypothesize that relative hypomethylation of* Olfr151* in F0 sperm may lead to inheritance of the hypomethylated* Olfr151* in F1 Main Olfactory Epithelium (MOE) and F1 sperm, creating an inheritance cascade.” However, the epigenetic mark was found in the sperm but not in the MOE of F1 mice. Noting that DNA methylation and histone modifications are known to be dependent on each other, the authors suggested that changes in the methylation pattern in sperm DNA might have resulted in histone modifications around the olfactory gene in MOE DNA.

The literature on effects of experimental exposure upon DNA methylation in rodent oocytes is less abundant compared with that on effects induced in sperm. Two papers report undermethylation of maternally imprinted genes in oocytes exposed to bisphenol-A either in vivo [164] or in culture [165]. In addition to the challenge posed by working with a little number of cells, experimental studies on female-mediated epigenetic inheritance also face the difficulty of strictly distinguishing a mechanism of epigenetic inheritance via the gametes from other mechanisms of epigenetic inheritance, such as those based on adverse uterine environment or lactation-mediated effects. This issue emerged, for example, in a recent paper [158] showing functional alterations of methylation patterns in the* Lep* and* Pparα* metabolic genes in oocytes of mice treated for 12 weeks with a high-fat diet, as well as in the liver cells of their offspring.

## 4. Discussion 

DNA methylation is a life-essential process that modulates gene expression and drives cell differentiation in multicellular organisms. Synergistically with other epigenetic mechanisms, it allows cells and organisms to adapt to external changes, in a timely way that mutational mechanisms could never meet. As such, DNA methylation is unsurprisingly sensitive to external stimuli. At the same time and in contrast to mutations, DNA methylation changes are reversible. This duality poses a challenge to researchers who aim to establish possible links between environmental exposure and epigenetic changes that may have a long-lasting impact on cell function and ultimately on health. Cancer, in all its forms, is the most typical example of a disease associated with aberrant epigenetics, which may be triggered by environmental exposure [2, 176], but ample evidence exists where erroneous epigenetic marks also play prominent roles in neurological disorders such as Alzheimer's disease, autoimmune diseases such as rheumatoid arthritis, and cardiovascular diseases, among others [2]. The path of environmental epigenetics will necessarily have to move from initially sparse association studies towards causal relationships supported by biological plausibility.

The plasticity of the human epigenome and the difficulty to sort out major environmental effects from “background noise” can be appreciated from the studies showing a seasonality and weather influence on some DNA methylation biomarkers analyzed in recent human biomonitoring studies [177, 178] or the findings of genome-wide analyses that showed an influence of long-term shiftwork on DNA methylation at several loci [179–182]. These latter studies, prompted by the evidence of an association between exposure to light at night, circadian rhythms, and cancer risk, demonstrated indeed methylation changes in many cancer-relevant genes and pathways, but they need to be confirmed by independent replication in larger samples and supported by fundamental mechanistic research, before any firm conclusion can be drawn.

In addition, the fact that interindividual variation in methylation may also be a consequence of DNA sequence polymorphisms that result in methylation quantitative trait loci should not be overlooked. Teh and coworkers [183] have investigated the genotypes and DNA methylomes of 237 neonates and found some 1500 punctuate regions of the methylome highly variable across individuals, termed variably methylated regions (VMRs), against a homogeneous background. The best explanation for 75% of VMRs was the interaction of genotype with different in utero environments, including maternal smoking, maternal depression, maternal BMI, infant birth weight, gestational age, and birth order. A prevalence of genetic over environmental determinants of interindividual variation of CpGs methylation has been recently reported in large Scottish and Australian cohorts [184].

Finally, age is expected to be a major variable affecting the DNA methylation profiles in different tissues. In fact, recent studies aimed at exploring the importance of epigenetic changes to the ageing process highlighted age-signatures of DNA methylation [185–187].

One of the problems in drawing an overall pattern from published literature on environmental epigenetic effects is due to the heterogeneity of detection methods and approaches. Several different methods have been developed for DNA methylation analysis and their advantages and drawbacks are discussed in excellent, recent reviews [188–191]. We have witnessed in a short time the passage from the analysis restricted to single specific regions to a global and genome-wide scale. Even if, on purely theoretical considerations, the ideal choice would point at a technique able to measure the entire methylome at a single-base-pair resolution in a particular cell system, researchers have to face other issues related to time- and cost-effectiveness and make reasonable compromises with their own scientific questions and the technology available. By and large, cost-affordable technologies are limited in their sensitivity to DNA methylation detection, like those relying on the global immunostaining of the 5-mCs or like pyrosequencing that analyzes only a limited amount of informative cytosines [192, 193]. On the other hand, technologies based on high-resolution methylation arrays [194] are able to measure countless sequences across the genome but are costly and demand sophisticated bioinformatics. The methylation analysis at targeted genes, like those imprinted, involved in some metabolic pathway, or supposedly metastable, and/or in repetitive elements (transposonic or not) is a frequently used approach in environmental epigenetics. Interestingly, it is emerging that repetitive elements, such as* Alu* and* LINE-1*, which were initially chosen simply as a proxy of the global methylation level due to their abundance throughout the genome, respond to environmental stress in a sequence-specific manner and have to be considered as separate entities [96, 98, 120, 195]. An international methodological standardization and harmonization effort would contribute to reaching more solid evidence on the epigenetic impact of environmental stressors. It certainly represents a Herculean task as the human haploid DNA methylome contains approximately 30 million CpGs that exist in a methylated, hydroxymethylated, or unmethylated state.

Notwithstanding such difficulties, environmental epigenetics may become a potent concept to fully assess the impact of the exposome on human health [196]. In particular, the notion that, in mammals, tissue differentiation is mainly established during prenatal life, and fundamental DNA methylation changes occur in preimplantation embryo and during gonadal differentiation, may support the hypothesis of prenatal origin of adult-onset diseases. To push science forward, epidemiological mother-child cohort studies and maternal exposure assessment will be instrumental.

Also the bases of reproductive health are founded during prenatal life, with primordial germ cell differentiation and gonad development, although the process of gametogenesis will only be completed after puberty. This means that multiple exposure windows must be considered to assess possible environmental effects on the gamete genetic and epigenetic integrity.

The results of the studies in rodents that we have described in the previous section show that DNA methylation in germ cells can be altered by many different kinds of exposure during the fetal as well as the adult life. Still, these studies suffer of some limitations: more data are available on the male than on the female germline, and only few of them carried out the analyses at the most informative genome scale, addressed the functional impact of epigenetic changes on the gene expression level and related cell pathways, and took into consideration dose-effect relationships. Nevertheless, their results are very important because they establish proof of principle demonstration that a variety of exogenous stressors may alter DNA methylation at developmentally important imprinted or metabolic genes.

As a target of environmental exposure, the germline meets a double risk, of compromising the reproduction capacity of the exposed individual and transmitting possible damage to the following generation. Some of the studies in rats and mice indeed showed that treatment induced not only DNA methylation changes in sperm but also phenotype alterations in the sired offspring. These observations are consistent with the notion that DNA methylation profiles of the gametes are not completely reset after fertilization but can be partly transmitted across generations. Actually, few experiments tested this notion in the specific conditions, with some showing apparent inheritance of gamete methylation [156, 168], while others not showing the same result [152]. Nevertheless, several authors agree in pointing out that direct transmission of methylation changes is not the only mechanism through which altered sperm methylation might affect the offspring phenotype and that sustained alterations of transcriptional regulatory networks early in development may likely result from a complex interplay between DNA methylation changes, chromatin modifications, and other epigenetic mechanisms. One implication of epigenetic inheritance systems is that they provide a potential mechanism by which parents could transfer information to their offspring about the environment they experienced. In other words, mechanisms exist that could allow organisms to “inform” their progeny about prevailing environmental conditions.

In some of the experimental studies [162, 163, 168, 169], changes of DNA methylation in the germline and somatic cells of exposed animals were compared. On the basis of the few available data, it is not possible to draw any general conclusion, but, much more than for induced genetic changes, it is conceivable that each cell type, with its own transcriptional program, would be specifically affected at an epigenetic level. This consideration poses a problem when data on induced epigenetic changes in the germline of experimental rodents want to be related with human biomonitoring data.

In fact, as previously shown, whereas the database on environmental factors impinging on DNA methylation in human leukocytes is already abundant, very few data exist on the variables affecting DNA methylation in human germ cells, even in the most easily accessible sperm. Acknowledging the limitation of a comparison between two large, but independent, studies, carried out in different cohorts, the increase of* LINE-1* methylation reported in blood cells in association with perfluorooctane sulfonate (PFOS) serum level [95], and the lack of an association between PFOS serum level and* LINE-1* methylation in sperm [96] exemplifies the difficulty of any extrapolation between somatic and germline environmental epigenetics.

Much more fruitful has been until now the field of male reproductive clinical epigenetics [35, 36]. The review of data showing DNA methylation and other epigenetic changes in the sperm of subfertile patients was out of the scope of this paper. However, these data are important also for reproductive environmental epigenetics because they seem to indicate a functional significance of DNA methylation changes in the male germline. At the same time, they evidence the need to conduct specific epigenetic analyses on the sperm of men exposed to reproductive toxicants, with the awareness that their PBLs could not surrogate the relevant target cells. Recently, the entire methylome of human sperm has been analyzed at high resolution thanks to the most advanced technologies [127, 197, 198]. While this dataset will be consolidated by repeated analyses and the degree of interindividual variation will be assessed, it will provide an essential reference for future studies on the impact of environmental stressors.

From an overall assessment of the current database on human somatic environmental epigenetics, rodent germline epigenetic toxicological studies, and the most environmentally relevant human reprotoxic agents, a priority list of environmental stressors on which directing future human sperm epigenetic biomonitoring studies might be proposed: dysmetabolism as a consequence of environmental and genetic factors, including their possible interactions, endocrine disrupting compounds, and major lifestyle toxicants like tobacco smoke and alcohol. In addition, emphasis should be on prenatal exposure, and mother child cohorts should be studied more actively. Finally, prospective, long-term, multigeneration follow-up surveys should be possibly set up to take into account grandparental effects.

## Figures and Tables

**Table 1:** Selected epidemiological studies on the effects of environmental chemical exposures on human DNA methylation.

Exposure (chemical)	Study population	Target tissue	Target DNA region	Method	DNA methylation changes	Reference
Metals						

Arsenic (As)	294 adults, India	PBL	Global	Methyl acceptance assay	Hypermethylation	[45]
	64 adults, India	PBL	Global	Methyl acceptance assay	Hypermethylation	[46]
	320 adults, Bangladesh	PBL	Global	Methyl acceptance assay	Hypermethylation	[47]
	96 adults, India	PBL	*p16*, *p53 *	Bisulfite methyl specific PCR	Hypermethylation	[48]
	202 women, Argentina	PBL	*p16*, *MLH1*, and *LINE-1 *	Bisulfite PCR pyrosequencing	Hypermethylation at *p16* and *MLH1 *	[51]
	163 patients with arseniasis and 110 controls, China	PBL	*p16 *	Bisulfite methyl specific PCR	Hypermethylation	[49]
	16 individuals of which 8 were with arsenicosis, Mexico	PBL	Epigenome-wide	Arrays	Differential methylation patterns	[50]
	10 individuals with skin lesions and 10 controls, Bangladesh	PBL	Epigenome-wide	Arrays	6 differentially methylated sites including *RHBDF1 *	[53]
	38 patients with urothelial carcinoma, Taiwan	Tumor biopsies	*DAPK *	Bisulfite PCR sequencing	Hypermethylation	[52]
	28 patients with urothelial carcinoma, Taiwan	Tumor biopsies	Epigenome-wide	Arrays	Hypermethylation in *CTNNA2*, *KLK7*, *NPY2R*, *ZNF132*, and *KCNK17 *	[54]
	113 mother-child pairs, Bangladesh	PBL (maternal and umbilical cord samples)	*p16*, *p53*, *LINE-1*, and *Alu *	Bisulfite PCR pyrosequencing	Hypermethylation at *p16* and *LINE-1 *	[55]
	113 mother-child pairs, Bangladesh	PBL (maternal and umbilical cord samples)	Global *LINE-1*, *Alu *	Methyl incorporation assay; bisulfite PCR pyrosequencing; LUMA (Luminometric Methylation Assay)	Hypermethylation (sex-dependent)	[57]
	71 newborns, Thailand	PBL (cord blood)	Global *LINE-1* *p53 *	Global: total 5-mC by HPLC-MS; *LINE-1*: COBRA *p53*: methylation-specific restriction endonuclease digestion	Hypermethylation at *p53 *	[58]
	44 newborns, Bangladesh	PBL	Epigenome-wide	Arrays	Differential methylation in thousands of sites	[56]
	134 infants, USA	PBL	Epigenome-wide	Arrays	Hypermethylation at several loci	[59]
	newborns, Mexico	PBL	Epigenome-wide	Arrays	Differential methylation in thousands of sites	[60]

Chromium (Cr)	115 workers and 60 controls, China	PBL	Global	ELISA	Hypomethylation	[61]

Mercury (Hg)	131 dental professionals, USA	Buccal mucosa	*LINE-1*, *DNMT1*, *SEPW1*, and *SEPP1 *	Bisulfite PCR pyrosequencing	Hypomethylation at *SEPP1* in males	[63]

Lead (Pb)	517 elderly men, USA	PBL	*LINE-1*, *Alu *	Bisulfite PCR pyrosequencing	Hypomethylation at *LINE-1 *	[64]
	9 exposed individuals	PBL	*p16 *	Methylation-specific PCR and thermal denaturation	Hypermethylation	[65]

Cadmium (Cd)	202 women, Argentina	PBL	*LINE-1*, *p16*, and *MLH1 *	Bisulfite PCR pyrosequencing	Hypomethylation at *LINE-1 *	[66]
	17 mother-child pairs, USA	PBL (maternal and newborn)	Epigenome-wide	Arrays	Differential methylation at subsets of genes	[68]

Selenium (Se)	286 adults, Bangladesh	PBL	Global	Methyl acceptance assay	Hypomethylation	[67]

Hg, Pb, and Cd	43 women undergoing IVF, USA	PBL	Epigenome-wide	Arrays	Hg: hypermethylation at *GSTM1*; Pb: hypomethylation at *COL1A2 *	[62]

Air pollution (including PM and PAH)	48 adult nonsmokers, Belgium	PBL	Global	HPLC	Hypomethylation	[70]
	718 elderly individuals, USA	PBL	*LINE-1*, *Alu *	Bisulfite PCR pyrosequencing	Hypomethylation at *LINE-1 *	[71]
	706 elderly individuals, USA	PBL	*LINE-1*, *Alu *	Bisulfite PCR pyrosequencing	Hypomethylation	[72]
	777 elderly individuals, USA	PBL	*F3*, *IFN-γ*, *IL-6*, *TLR-2*, and *ICAM-1 *	Bisulfite PCR pyrosequencing	*F3*, *ICAM-1*, and *TLR-2* hypomethylation; *IFN-γ* and *IL-6* hypermethylation	[73]
	776 elderly individuals, USA	PBL	*F3*, *ICAM-1*, *IFN*-*γ*, *IL-6*, *TLR-2*, *CRAT*, *GCR*, *iNOS*, and *OGG1 *	Bisulfite PCR pyrosequencing	Differential methylation at *TLR2*, *GCR*, *TLR2*, *F3*, and *IL6 *	[74]
	63 steel workers, Italy	PBL	*LINE-1*, *Alu*, *iNOS* *p16*, *p53*, *hTERT*, and *APC*, *RASSF1A *	Bisulfite PCR pyrosequencing	Hypomethylation at *iNOS*, *p53*, and *RASSF1A*; hypermethylation at *APC*, *p16 *	[81–83]
	49 nonsmoking coke-oven workers and 43 controls, Poland	PBL	*p16*, *p53*, *IL-6*, *HIC1*, *LINE-1*, and *Alu *	Bisulfite PCR pyrosequencing	Hypermethylation at *LINE-1*, *Alu*, and *IL-6*; hypomethylation at *p53 *	[84]
	60 truck drivers and 60 controls, China	PBL	Tandem repeats *Satα*, *NBL2*, and *D4Z4 *	Bisulfite PCR pyrosequencing	Hypomethylation at *Satα* and *NBL2 *	[85]
	120 male workers, Italy and China	PBL	*LINE-1* subfamilies (*L1HS*, *L1PA5*, *L1PA2*, and *L1Ta*); *Alu* subfamilies (*AluSx*, *AluYb8*, and *AluYd6*); *HERV* subfamilies (*MLT1D*, *ERV1*, and *ERV9*)	Bisulfite PCR pyrosequencing	Differential methylation	[87]
	67 industrial workers, 65 residents, and 45 rural controls, Thailand	PBL	*LINE-1*, *Alu*, *p16*, *p53*, *HIC1*, and *IL-6 *	Bisulfite PCR pyrosequencing	Hypomethylation at *p53*, *IL-6*, and *LINE-1 *	[88]
	78 gasoline filling attendants, 77 urban traffic officers, 57 control office workers, Italy	PBL	*LINE-1*, *Alu*, *p15*, and *MAGE1 *	Bisulfite PCR pyrosequencing	Hypomethylation at *LINE-1*, *Alu*, and *MAGE1*; hypermethylation at *p15 *	[89]
	78 gasoline filling attendants, 58 control office worker, Italy	PBL	Global	High-resolution GC-MS	Hypomethylation	[90]
	158 petrochemical workers and 50 control office workers, Bulgaria	PBL	*LINE-1*, *Alu*, *p15*, and *MAGE1 *	Bisulfite PCR pyrosequencing	Hypomethylation at *LINE-1* and *p15 *	[91]
	141 asthmatic children and 70 controls, USA	Treg cells from PBL	*Foxp3 *	?	Hypermethylation	[75]
	940 children, USA	Buccal cells	*NOS1*, *NOS2A*, and *NOS3 *	Bisulfite PCR pyrosequencing	Differential methylation at *NOS2A* and *NOS3 *	[76]
	56 mother-child pairs, USA	PBL (cord blood)	*ACSL3 *	Methylation-specific PCR	Hypermethylation	[79]
	164 mother-child pairs, USA	PBL (cord blood)	Global	ELISA	Hypomethylation	[78]
	53 mother-child pairs, USA	PBL (cord blood)	*IFNγ* and *IL-4 *	Bisulfite PCR sequencing	Hypermethylation at *IFNγ*	[80]

POPs	70 Inuits, Greenland	PBL	*LINE-1*, *Alu *	Bisulfite PCR pyrosequencing	Hypomethylation at *Alu *	[92]
	86 adults, South Korea	PBL	*LINE-1*, *Alu *	Bisulfite PCR pyrosequencing	Hypomethylation at *Alu *	[93]
	358 mother-child pairs, USA	PBL (cord blood and at 9 years)	*LINE-1*, *Alu *	Bisulfite PCR pyrosequencing	Hypomethylation at *Alu *	[98]

Bisphenol A	43 women undergoing IVF, USA	PBL	Epigenome-wide	Arrays	Hypomethylation at the *TSP50 *	[62]
	46 preadolescent girls, Egypt	Saliva	Epigenome-wide	Arrays	Hypomethylation at several loci	[94]

PFASs	685 adults, USA	PBL	*LINE-1 *	Bisulfite PCR pyrosequencing	Hypermethylation	[95]
	262 fertile men, Greenland/Poland/Ukraine	Sperm	Global; *LINE-1*, *Alu*, and *Satα*	Flow cytometry immunodetection of 5-mC; bisulfite PCR pyrosequencing	No consistent changes	[96]
	30 mother-child pairs, USA	PBL (cord blood)	Global	ELISA	Hypomethylation	[97]

EDs	192 mother-child pairs, Spain	Placenta	*LINE-1* subfamilies (*L1HS*, *L1PA5*, *L1PA2*, and *L1Ta*); *Alu* subfamilies (*AluSx*, *AluYb8*, and *AluYd6*); *HERV* subfamilies (*MLT1D*, *ERV1*, and *ERV9*)	Bisulfite PCR pyrosequencing	*AluYb8* hypomethylation in boys	[99]

Tobacco smoke	177 adults, Germany	PBL	Epigenome-wide	Arrays	Hypomethylation at *F2RL3 *	[103]
	374 adults, Italy	PBL	Epigenome-wide	Arrays	Hypomethylation at *F2RL3*, *AHRR*, and other loci	[105]
	261 adults, Italy	PBL	*AHRR* 6p21 (1 locus) 2q37 (2 loci)	Bisulfite PCR pyrosequencing	Differential methylation	[111]
	3,588 adults, Germany	PBL	*F2RL3 *	MALDI-TOF MS	Hypomethylation	[106]
	399 African American youths, USA	PBL	Epigenome-wide	Arrays	Hypomethylation at *AHRR *	[109]
	107 African American young men, USA	PBL	Epigenome-wide	Arrays	Hypomethylation at *AHRR *	[110]
	111 adult African American women, USA	PBL	Epigenome-wide	Arrays	Differential methylation at 910 loci; hypomethylation at *AHRR* and *GPR15 *	[112]
	348 mother-child pairs, USA	Child buccal cells	*LINE-1*, *AluYb8 *	Bisulfite PCR pyrosequencing	Hypomethylation at *AluYb8 *	[113]
	272 mother-child pairs, USA	Child buccal cells	Epigenome-wide	Arrays	Differential methylation in 8 genes; Hypermethylation at *AXL* and *PTPRO *	[113]
	173 mother-child pairs, USA	Child buccal cells	*AXL *	Bisulfite PCR pyrosequencing	Hypermethylation	[114]
	527 mother-asthmatic child pairs, USA	Child PBL	Epigenome-wide	Arrays	Differential methylation at 19 loci	[115]
	36 mother-child pairs (18 nonsmokers and 18 smokers), USA	Placenta	Epigenome-wide	Arrays	Differential methylation at several loci	[117]
	1,062 mother-child pairs, Norway	PBL (cord blood)	Epigenome-wide	Arrays	Differential methylation in 10 genes including *AHRR*, *CYP1A1*, and *GFI1 *	[118]
	418 mother-child pairs, USA	PBL (cord blood)	DMRs of *IGF2* and *H19 *	Bisulfite PCR pyrosequencing	*IGF2* DMR differential methylation	[119]
	380 mother-child pairs, USA	PBL (cord blood)	*LINE-1*, *Alu *	Bisulfite PCR pyrosequencing	Hypermethylation at *AluYb8 *	[120]
	206 mother-child pairs, USA	Placenta	Epigenome-wide	Arrays	Hypermethylation at *RUNX3 *	[121]
	132 mother-child pairs, Canada	PBL (adolescents)	Epigenome-wide	Arrays	Hypermethylation at *MYO1G*; hypomethylation at *CNTNAP2 *	[122]

**Table 2:** Synopsis of papers reporting effects of experimental treatments on DNA methylation of rodent male or female germ cells (when additional tissues were analyzed, they are specified).

Exposure	Exposed animals	Dose and time of exposure	Target tissue	Target DNA region	Method	DNA methylation changes	Reference
Flutamide	Rats	In utero exposure: i.p. injection of 10 mg/kg/day, between day 8 and day 15 of gestation	Testis cells of 6-day-old animals; sperm of adult animals	*LPLase *	Bisulfite PCR sequencing	No detectable DNA methylation changes	[160]

Procymidone	Rats	In utero exposure: i.p. injection of 100 mg/kg/day, between day 8 and day 15 of gestation	Testis cells of 6-day-old animals	*LPLase *	Bisulfite PCR sequencing	No detectable DNA methylation changes	[160]

Vinclozolin	Rats	In utero exposure: i.p. injection of 100 mg/kg/day, between day 8 and day 15 of gestation	Testis cells of 6-day-old animals	*LPLase *	Bisulfite PCR sequencing	No detectable DNA methylation changes	[160]

Vinclozolin	Rats	In utero exposure: i.p. injection of 100 mg/kg/day, between day 8 and day 15 of gestation	Testis cells of 6-day-old animals	Multiple, unspecified	Methylation-specific restriction Endonuclease digestion	Altered methylation detected at multiple sequences involving both hypo- and hypermethylation events	[159]

Vinclozolin	Mice	In utero exposure: i.p. injection of 50 mg/kg/day, between day 10 and day 18 of gestation	Sperm, plus tail, liver, and skeletal muscle cells in adult animals	*H19*, *Meg3*, *Mest*, *Snrpn*, and *Peg3 *	Bisulfite PCR pyrosequencing	In sperm, highly significant *H19* and *Meg3* hypomethylation and *Mest*, *Snrpn*, and *Peg3* hypermethylation; less evident effects in somatic tissues	[161]

Dioxin	Mice	In utero exposure: i.p. injection of 2 or 10 ng/kg/day between day 9 and day 19 of gestation	Sperm, plus liver and skeletal muscle cells in adult animals	*Snrpn*, *Peg3*, and *Igf2r *	Bisulfite PCR pyrosequencing	No detectable DNA methylation changes in sperm; in liver and muscle cells significant increases of methylation in *Igf2r *	[163]

Methoxychlor	Mice	In utero exposure: i.p. injection of 10 mg/kg/day, between day 10 and day 18 of gestation	Sperm, plus tail, liver, and skeletal muscle cells in adult animals	*H19*, *Meg3*, *Mest*, *Snrpn*, and *Peg3 *	Bisulfite PCR pyrosequencing	Significantly decreased methylation of *H19* and *Meg3* and significantly increased methylation of *Mest*, *Snrpn*, and *Peg3* in sperm; no effect in somatic tissues	[162]

Ethanol	Mice	In utero exposure: p.o. administration of 0.5 g/kg/day between day 10 and day 18 of gestation	Sperm, plus tail, liver, skeletal muscle, and brain cells in adult animals	*H19*, *Meg3*, *Mest*, *Snrpn*, and *Peg3 *	Bisulfite PCR pyrosequencing	Highly significant 3% decrease in the number of methylated CpGs of *H19*; no effect in the other genes and in somatic tissues	[168]

Folate-deficient diet	Mice	In utero exposure: treatment of dams with folate-deficient diet from two weeks before mating throughout gestation and lactation	Sperm	Epigenome-wide	Arrays	57 genomic regions had altered methylation profiles in sperm from males exposed to folate-deficient diet; both hypo- and hypermethylation were observed; methylation differences observed in promoter regions of genes implicated in development and with functions in the central nervous system, kidney, spleen, digestive tract, and muscle tissue, and of genes associated with diabetes, autoimmune diseases, neurological diseases, autism, schizophrenia, and cancer	[154]

Undernourishment	Mice	In utero exposure: nutritional restriction between day 12.5 and day 18.5 of gestation	Sperm	Global; epigenome-wide	Mass spectrometry; arrays	166 differentially methylated regions, of which 111 were hypomethylated and 55 hypermethylated in undernourished relative to control mice; the bisulfite pyrosequencing validation confirmed 17/24 hypomethylated and 0/8 hypermethylated regions	[152]

Methoxychlor	Mice	Adult exposure: i.p. injection of 10 mg/kg/day for 8 consecutive days	Sperm, plus tail, liver, and skeletal muscle cells in adult animals	*H19*, *Meg3*, *Mest*, *Snrpn*, and *Peg3 *	Bisulfite PCR pyrosequencing	Significantly decreased methylation of *Meg3* and significantly increased methylation of *Mest*, *Snrpn*, and *Peg3* in sperm; no effect in somatic tissues	[162]

Ethanol	Mice	Adult exposure: p.o. administration of 5.9 g/kg/day for 29 days over a period of 5 weeks	Sperm	*H19*, *Rasgrf1 *	Bisulfite PCR pyrosequencing	No detectable DNA methylation changes	[169]

Fenvalerate	Mice	Adult exposure: p.o. administration of 10 mg/kg/day for 30 days	Sperm	Epigenome-wide	Arrays	Significant *Hap1* hypomethylation; significant *Ace*, *Foxo3a*, *Nr3c2*, *Pml*, and *Ptgfrn* hypermethylation	[172]

Sodium fluoride	Mice	Adult exposure: p.o. administration of 100 mg/L in drinking water for 35 days	Sperm, plus liver cells in adult animals	*H19*, *Rasgrf1*, *Peg3*, and *Line-1;* global	Bisulfite PCR sequencing; ELISA	No detectable DNA methylation changes	[173]

Tamoxifen	Rats	Adult exposure: p.o. administration of 0.4 mg/kg/day 5 days a week for 60 days	Sperm	*Igf2-H19;* global	Bisulfite PCR sequencing; flow cytometry after immunostaining with 5-methyl cytosine antibody	Significantly reduced methylation at *Igf2-H19;* global methylation level unaffected	[171]

Tamoxifen	Rats	adult exposure: p.o. administration of 0.4 mg/kg/day 5 days a week for 60 days	Sperm	*Dlk1*, *Plagl1*, *Peg5*, *Peg3*, *Igf2r*, *Grb10*, *Kcnq1*, *Ascl2*, and *Cdkn1c *	Bisulfite PCR sequencing	No detectable DNA methylation changes	[170]

Bisphenol A	Rats	Neonatal exposure: 5 daily subcutaneous injections of 0.4 mg/kg/day BPA starting one day after birth	Sperm	*Igf2-H19 *	Bisulfite PCR sequencing	Significant hypomethylation at the *H19* ICR	[166]

Particulate air pollution	Mice	Adult exposure: 3 or 10 weeks of exposure to ambient air in polluted sites Sperm sampling either immediately or 6 weeks after the end of 10-week exposure	Sperm	Global	Cytosine extension assay and methyl acceptance assay	Significantly increased global methylation in 10-week exposed samples, which persisted after exposure interruption; methylation changes abolished by use of air filters	[167]

High-fat diet	Mice	Adult exposure: 10-week exposure to high-fat diet from 5 weeks of age	Testis cells; elongated spermatids	Global	ELISA; semiquantitative immunohistochemistry of testis sections with anti-5-mC antibody	About 25% reduction in global methylation in both whole testicular cells and spermatids	[149]

High-fat diet plus streptozotocin subdiabetogenic treatment	Mice	Adult exposure: 13-week exposure to high-fat diet, from 3 weeks of age, plus streptozotocin i.p. injection at 12 weeks of age	Sperm	Epigenome-wide	Arrays	Paternal prediabetes altered overall methylome patterns in sperm, with a large portion of differentially methylated genes overlapping with that of pancreatic islets in offspring	[156]

Low-protein diet	Mice	Adult exposure: 9-week exposure to low-protein diet from 3 weeks of age	Sperm	*Pparα;* epigenome-wide	Bisulfite PCR sequencing; arrays	No effects of diet on *Pparα* methylation in sperm; overall, sperm cytosine methylation patterns were largely conserved under various dietary regimes	[153]

Olfactory fear conditioning	Mice	Adult exposure to acetophenone	Sperm	*Olfr151 *	Bisulfite PCR sequencing	Hypomethylation	[175]

Streptozotocin diabetogenic treatment	Mice	Adult exposure: single i.p. injection of 230 mg/kg streptozotocin, 15, 25, or 35 days before oocyte collection after superovulation	Oocytes	*H19*, *Peg3*, and *Snrpn *	COBRA	Evident demethylation was observed in the methylation pattern of *Peg3* DMR on day 35 after treatment; the methylation patterns of *H19* and *Snrpn* DMRs were not significantly altered by maternal diabetes	[157]

High-fat diet	Mice	Adult exposure: 12 weeks of feeding with high-fat diet prior to oocyte collection by superovulation	Oocytes	*H19*, *Mest*, *Peg3*, *Igf2r*, *Snrpn*, *Pparα*, and *Lep *	COBRA	DNA methylation of imprinted genes was not altered; the *Pparα* methylation level was significantly decreased and the *Lep* methylation level was significantly increased in high-fat diet fed mice compared with control mice	[158]

Zinc-deficient diet	Mice	Adult exposure: 5 days of feeding with zinc-deficient diet prior to oocyte collection by superovulation	Oocytes	Global	Immunocytochemistry with anti-5-mC antibody	DNA methylation was reduced to about 60% of control levels in zinc-deficient oocytes	[155]

Bisphenol A	Mice	Neonatal exposure: 20 or 40 *μ*g/kg b.w. either by daily hypodermal injections from postnatal day 7 to postnatal day 14 or by intraperitoneal injections administered each fifth day between postnatal days 5 and 20, prior to collection of ovarian oocytes	Oocytes	*H19*, *Igf2r*, and *Peg3 *	Bisulfite PCR sequencing	Significant dose-dependent reduction of methylation in *Igf2r*, *Peg3* genes*;* no effect on *H19 *	[164]

Bisphenol A	Mice	In vitro exposure to 3 or 300 nM during 12 days of follicle culture from preantral to antral stage	Oocytes	*H19*, *Mest*, *Igf2r*, and *Snrpn *	Bisulfite PCR pyrosequencing	Significantly decreased methylation at the low, but not at the high, BPA dose, in *Mest*, *Igf2r*, and *Snrpn* genes; no effect on *H19 *	[165]

## Abbreviations

ABCA1: ATP-binding cassette, subfamily A (ABC1), member 1
ACE: Angiotensin I-converting enzyme
ACSL3: Acyl-CoA synthetase long-chain family member 3
AHRR: Aryl hydrocarbon receptor repressor
APC: Adenomatous polyposis coli
ASCL2: Achaete-scute family bHLH transcription factor 2
AXL: AXL receptor tyrosine kinase
BMI: Body mass index
CDKN1C: Cyclin-dependent kinase inhibitor 1C
CNTNAP2: Contactin associated protein-like 2
COBRA: Combined bisulfite restriction analysis
COL1A2: Collagen, type I, alpha 2
CRAT: Carnitine O-acetyltransferase
CTCF: CCCTC-binding factor (zinc finger protein)
CTNNA2: Catenin (cadherin-associated protein), alpha 2
CYP1A1: Cytochrome P450, family 1, subfamily A, polypeptide 1
DAPK: Death-associated protein kinase
DLK1: Delta-like 1 homolog
DMR: Differentially methylated region
DNMT1: DNA (cytosine-5-)-methyl transferase 1
EDs: Endocrine disruptors
ELISA: Enzyme linked immunosorbent assay
F2RL3: Coagulation factor II (thrombin) receptor-like 3
F3: Coagulation factor III
FOXO3A: Forkhead box O3
FOXP3: Forkhead box transcription factor 3
GC-MS: Gas chromatography-mass spectroscopy
GCR: Glucocorticoid receptor
GFI1: Growth factor independent 1 transcription repressor
GNASAS: GNAS antisense RNA
GPR15: G protein-coupled receptor 15
GRB10: Growth factor receptor-bound protein 10
GSTM1: Glutathione S-transferase mu 1
H19: Imprinted maternally expressed transcript (nonprotein coding)
HAP1: Huntingtin-associated protein 1
HERV: Human endogenous retrovirus
HIC1: Hypermethylated in cancer 1
HPLC-MS: High performance liquid chromatography-mass spectrometry
hTERT: Human telomerase reverse transcriptase
ICAM-1: Intercellular adhesion molecule 1
ICR: Imprinting control center
IFN*γ*: Interferon gamma
IGF2: Insulin-like growth factor 2
IGF2R: Insulin-like growth factor 2 receptor
IL-4: Interleukin-4
IL-6: Interleukin-6
IL-10: Interleukin-10
iNOS: Inducible nitric oxide synthase
IVF: In vitro fertilization
KCNK17: Potassium channel, subfamily K, member 17
KCNQ1: Potassium voltage-gated channel, KQT like subfamily, member 1
KLK7: Kallikrein-related peptidase 7
LBW: Low birth weight
LEP: Leptin
LINE-1: Long interspersed nuclear element 1, retrotransposable element 1
LPLASE: Lysophospholipase
5-mC: 5-methyl cytosine, 5-methyl deoxycytidine
MAGE1: Melanoma antigen family A, 1
MALDI-TOF MS: Matrix-assisted laser desorption ionization time-of-flight mass spectrometry
MEG3: Maternally expressed 3 (nonprotein coding)
MEST: Mesoderm specific transcript
MLH1: MutL homolog 1
MYO1G: Myosin IG
NNAT: Neuronatin
NOS1: Nitric oxide synthase 1
NOS2A: Nitric oxide synthase 2A
NOS3: Nitric oxide synthase 3
NPY2R: Neuropeptide Y receptor Y2
NR3C2: Nuclear receptor subfamily 3, group C, member 2
OGG1: 8-Oxoguanine DNA glycosylase 1
OLFR151: Olfactory receptor 151
p15: Cyclin-dependent kinase inhibitor 2B
p16: Cyclin-dependent kinase inhibitor 2A
p53: Tumor protein p53
PAHs: Polycyclic aromatic hydrocarbons
PBL: Peripheral blood lymphocytes
PCBs: Polychlorinated biphenyls
PCR: Polymerase chain reaction
PEG3: Paternally expressed 3
PEG5: Paternally expressed 3 (alias* NNAT*: neuronatin)
PEG10: Paternally expressed 10
PFASs: Perfluoroalkyl substances
PGC: Primordial germ cell
PLAGL1: Pleomorphic adenoma gene-like 1
PM: Particulate matter
POPs: Persistent organic pollutants
PPAR*α*: Peroxisome proliferator-activated receptor alpha
PTGFRN: Prostaglandin F2 receptor negative regulator
PTPRO: Protein tyrosine phosphatase, receptor type, O
RASGRF1: Ras protein-specific guanine nucleotide-releasing factor 1
RASSF1A: Ras association (RalGDS/AF-6) domain family member 1
RHBDF1: Rhomboid family member 1
RUNX3: Runt-related transcription factor 3
SEPP1: Selenoprotein P, plasma, 1
SEPW1: Selenoprotein W, 1
SGCE: Sarcoglycan, epsilon
SNRPN: Small nuclear ribonucleoprotein polypeptide N
TLR-2: Toll-like receptor 2
TSP50: Testes-specific protease 50
ZNF132: Zinc finger protein 132.
